# Psychological disturbances of survivors throughout COVID-19 crisis: a qualitative study

**DOI:** 10.1186/s12888-020-03009-w

**Published:** 2020-12-17

**Authors:** Yaser Moradi, Farzin Mollazadeh, Parivash Karimi, Keyvan Hosseingholipour, Rahim Baghaei

**Affiliations:** Patient Safety Research Center, Clinical Research Institute, School of Nursing and Midwifery, Urmia University of Medical Sciences, Urmia, Iran

**Keywords:** COVID-19, Psychological disturbances, Survivors, Qualitative research, Phenomenology

## Abstract

**Background:**

In addition to physical problems, patients with COVID-19 suffer from considerable stress throughout the disease crisis. It is important to address mental health needs and not to ignore the psychological dimension in this group of patients. In this regard, the first practical step is to have a clear understanding of patients’ psychological issues. Therefore, this study aimed to explore the psychological disturbances of COVID-19 survivors throughout the disease crisis.

**Method:**

This qualitative study was conducted using a phenomenological approach through 14 individual semi-structured in-depth interviews with patients recovered from COVID-19. Data were analyzed using Colaizzi’s seven-step method.

**Results:**

Three themes of “living in limbo”, “psychological distress behind the wall” and “psychological burden of being a carrier” were extracted as the psychological disturbances of COVID-19 survivors throughout the disease crisis.

**Conclusion:**

This study portrayed a better understanding of psychological disturbances of COVID-19 survivors throughout the disease crisis based on their lived experiences. Given the ambiguity in the time of the disease eradication and its continuing course, a deep understanding of these experiences in the current critical situation can help healthcare officials to make appropriate decisions and take measures to assess and identify psychological traumas and perform interventions to improve the mental state of these patients.

## Background

The COVID-19 pandemic is rapidly increasing throughout the world, with 235 countries affected until 15 October 2020 [1, 2]. On 11 March 2020, the WHO officially declared that the prevalence of COVID-19 has reached a global pandemic level. According to global statistics released by the WHO, the COVID-19 mortality rate is 7%. So far, more than 38 million cases have been confirmed and more than a million deaths have occurred [2].

COVID-19 is considered as a critical and stressful stage in patients’ lives [3, 4]. Throughout this crisis, patients suffer from considerable stress in addition to physical ailments [5] because acute infectious outbreaks have negative effects on their mental health in addition to potentially destructive effects on the physical health of patients, as observed in previous infectious epidemics like SARS and MERS [1, 6].

Patients with epidemic infections experience more psychological problems during outbreaks than other patients [7], such that even after treatment and discharge, they may suffer from varying degrees of stress disorders, anxiety, and long-term mental health problems. Studies on SARS survivors indicated persistent mental disorders such as post-traumatic stress disorder (PTSD) in 40% of survivors even after 3 years [1]. This highlights the need to address the mental health needs and not to ignore the psychological dimension in this group of patients [6].

The first practical step in achieving the above goal is to have a clear understanding of patients’ psychological issues [8]. Explaining and understanding the psychological disturbances of COVID-19 patients throughout and after the disease crisis could be very effective in expanding knowledge on how to better deal with possible future pandemics and prevent psychological trauma caused by similar pandemics by gaining insight into the current situation [9, 10]. Since people’s psychological experiences are the product of time and the interactive effects of individual and sociocultural factors, as well as a personal and unique experience [11], the most effective way to explain psychological disturbances of COVID-19 patients is using the lived experiences of patients who lived through and survived the disease. Therefore, this study aimed to explore the psychological disturbances of COVID-19 survivors throughout the disease crisis using a naturalistic view.

## Methods

### Study design

This was a descriptive phenomenological qualitative study based on the Colaizzi’s approach [12]. Considering the study question of “What are the psychological disturbances of COVID-19 survivors throughout the disease crisis?”, the researcher tried to describe the psychological disturbances of these people and highlight its nature based on their lived experiences.

### Participants and setting

Fourteen COVID-19 survivors in Urmia in northwestern Iran were selected using purposive sampling. The researcher first referred to the hospital admission and discharge office and prepared a list of the characteristics of all patients who had been discharged with a good general condition from the beginning of March 2020 to the end of May 2020. Then, the researcher started the sampling process. To achieve a wide range of experiences, participants were selected with a maximum variation of demographic characteristics such as age and gender (Table 1).

**Table 1:** Demographic characteristics of the study participants

Participant no.	Age (years)	Gender
P1	30	Male
P2	46	Male
P3	38	Female
P4	35	Male
P5	33	Female
P6	36	Male
P7	29	Female
P8	34	Female
P9	48	Male
P10	39	Male
P11	41	Female
P12	34	Female
P13	37	Female
P14	43	Male

Inclusion criteria consisted of (a) being COVID-19 survivor, (b) being hospitalized, (c) being isolated at home or nursing homes, (d) willingness to participate in the study and sharing experiences.

## Data collection

In-depth semi-structured individual interviews via phone calls were used to collect data. Interviews were conducted via phone calls to prevent any possible spread of the virus because it has been shown that patients recovered from COVID-19 can be still contagious for a certain amount of time [13, 14]. Then, the participants were asked to describe their experiences regarding the main questions of this study, as follows:

**“How did you feel when you found out you had COVID-19? Please tell us your experiences in this regard.”**

**“Describe your psychological disturbances and perceptions of the time you were hospitalized.”**

**“Tell us about your feelings and psychological state when you were in isolation.”**

The interviewer then advanced the interviews based on the participants’ responses toward a more in-depth examination of their experiences on the subject using probing questions such as “What do you mean? “, “Please explain more”, “Can you make your point clearer? “, “Why?”, and “How?”

The researcher recorded all the interviews with the consent of the participants. Each interview lasted about 40 min. The interviews continued until data saturation.

### Data analysis

Data were analyzed using Colaizzi’s seven-step method [12, 15]. In the first step, at the end of each interview and note-taking, recorded interviews were listened to several times and transcribed verbatim on paper and then typed into MS Word™ by two of the authors (FM, and PK). Then they were entered into the MAXQDA software 10.0 R250412 by three of the authors (YM, RB, and FM) for analysis, extract of concepts, and classification of data. Interview transcripts were read several times to understand the emotions and experiences of participants. In the second step, the significant information and statements related to the phenomenon were identified. In the third step, the concepts were extracted, and themes emerging from significant statements of each interview were identified such that a concept that expressed the core meaning of each statement of the participant’s thoughts was extracted. In the fourth step, the developed concepts were carefully studied and clustered based on the similarity of concepts. Thus, clustered themes were formed from the developed concepts. In the fifth step, the results were linked to describe the phenomenon, and more general categories were formed. In the sixth step, a comprehensive description of the phenomenon was presented. In the seventh step, the findings were verified by referring to each participant and asking about the findings.

### Rigor

In this study, the researcher tried to establish good communication with the participants, gain their confidence, and do the interviews in a comfortable atmosphere. Three of the randomly-selected interviews were also analyzed by the colleagues. Moreover, maximum variation was observed in selecting the participants to enhance the transferability of the findings to other situations or groups.

### Ethical considerations

At baseline, after obtaining approval of the Regional Committee for Medical Research Ethics (IR.UMSU.REC.1399.020), the researcher introduced himself and explained the study objectives to the participants. Then, he sent written informed consent image to the participants via messaging apps. The participants carefully read, signed and returned the image of the consent form to the researcher. The participants were assured that the information obtained would remain confidential. Moreover, after transcribing the interviews, all the recorded conversations were deleted.

### Findings

Of the 14 participants, seven were male and the rest were female. They were all in the 29–48 age range and their educational status ranged from being illiterate to having a bachelor’s degree. Most participants referred to the hospital with common symptoms of COVID-19 including fever, cough, muscle pain, and dyspnea. Three of the participants had been hospitalized in the Intensive Care Unit (ICU) without being intubated and the rest were hospitalized in the general wards. The length of hospital stay varied from 5 to 14 days.

Data analysis led to the emergence of 22 codes, 8 sub-themes, and 3 themes. Three themes of “living in limbo”, “psychological distress behind the wall”, and “psychological burden of being a carrier” were extracted as the psychological disturbances of COVID-19 survivors throughout the disease crisis (Table 2).

**Table 2:** Themes, subthemes, and codes obtained from data analysis

Themes	Subthemes	Codes	Frequency of the codes mentioned by participants
**Living in limbo**	**Confusion caused by new coronavirus infodemic**	Doubts about the accuracy of the information provided by the media	(P1)*2/ (P5)*3/ (P6)*2/ (P7)*1/ (P8)*2/ (P9)*1/ (P13)*1
		Being cluttered with information	(P3)*2/ (P7)*1/ (P6)*3/ (P10)*2/ (P14)*4
		Exacerbated anxiety by hearing conflicting news	(P2)*1/ (P3)*3/ (P5)*1/ (P13)*2
	**Confusion caused by vague prognosis**	Fear of recurrence	(P1)*1/ (P2)*2/ (P3)*2/ (P5)*2/ (P7)*1/ (P8)*1/ (P10)*2/ (P11)*2/ (P14)*1
		Fear of unpredictable complications	(P1)*1/ (P2)*1/ (P9)*1
		Fear of falling asleep and not waking up	(P1)*2/ (P6)*1/ (P13)*1/ (P14)*1
		Having nightmares	(P1)*1/ (P3)*1/ (P5)*1/ (P6)*1
	**A sense of imminent death**	Stress due to high mortality	(P1)*1/ (P2)*1/ (P3)*4/ (P4)*1/(P5)*1/ (P7)*1/ (P10)*2/ (P13)*2/ (P14)*1
		Feeling of approaching death	(P2)*1/ (P4)*1/ (P10)*1/ (P12)*1
		Being in a life-or-death situation	(P4)*1/ (P10)*1/ (P12)*1
**Psychological distress behind the wall**	**Missing others behind the COVID-19 wall**	Waiting for a normal day	(P6)*1/ (P9)*1/ (P13)*1
		Dull isolation days	(P1)*1/ (P6)*1/ (P9)*1/ (P13)*1
		Missing family	(P1)*1/ (P2)*1/ (P3)*1/ (P4)*2/(P5)*1/ (P6)*1/ (P8)*1/ (P9)*1/ (P11)*1/ (P12)*2/ (P13)*1/ (P14)*1
	**Psychological distress within the family**	Cold atmosphere of the family	(P3)*1/ (P8)*1/ (P12)*1
		Mental vulnerability of family members	(P2)*1/ (P6)*2/ (P8)*2/ (P10)*1
		Arguments	(P6)*1/ (P8)*1/ (P10)*1
**Psychological burden of being a carrier**	**Fear of transmitting the disease**	Fear of transmitting the disease to family members	(P1)*2/ (P2)*1/ (P3)*2/ (P4)*1/ (P5)*1/ (P6)*2/ (P8)*2/ (P9)*3/ (P10)*1/ (P11)*1/ (P13)*1/ (P14)*1
		Worrying about infecting other people	(P1)*1/ (P4)*1/ (P6)*1/ (P7)*2/ (P8)*1/ (P9)*2/ (P11)*1/ (P12)*1
	**Feeling rejected**	Staying away from others	(P3)*1/ (P10)*1/ (P14)*1
		Limited communication between the medical staff and the patient	(P1)*1/ (P4)*2/ (P5)*1/ (P11)*1/ (P14)*1
	**COVID-19 stigma**	Negative view of others	(P1)*1/ (P3)*1/ (P4)*2/ (P6)*2/ (P9)*12/ (P13)*2
		Annoying sympathies of others	(P1)*1/ (P4)*1/ (P5)*1/ (P11)*1/ (P14)*1

### Theme 1: living in limbo

Based on the participants’ experiences, the confusion caused by new coronavirus infodemic, confusion caused by vague prognosis, and a sense of imminent death created a state of uncertainty and turmoil in their lives. Participants described this experience as being trapping between life and death.

### Confusion caused by new coronavirus infodemic

Doubts about the accuracy of the information provided by the media, being cluttered with information, and exacerbated anxiety by hearing conflicting news led to confusion among patients. Participants described their experiences in this regard as follows.

*“I used to check the Internet whenever one of my family members or I got sick. Unfortunately, almost all the information on the Internet about this disease was full of suspicion and speculation. One website would write something and another website would write something else; and interestingly every TV channel would say something different about the disease. I really couldn’t figure out which one is true!” (Male patient, P6).*

*“We received a lot of conflicting information about the disease every day; for example, one would say take vitamin C, but another one would reject it and say ‘No, it is acidic and would exacerbate coughs’; one would say this is its vaccine, another one would say no it isn’t; what concerned me more was about what we finally should do with this disease!” (Female patient, P3).*

### Confusion caused by vague prognosis

Fear of recurrence and unpredictable complications had caused psychological disturbances in patients. The disturbance was mostly related to the concerns about the exacerbation of the disease and death. Participants described their experiences in this regard as follows.

*“I’m worried about whether someone that has had corona will be infected again; I searched the Internet for two or three hours to see what new articles say; I still have the stress; when I have 100% protection, will I be safe to go into the society!?” (Female patient, P5).*

*“Worries are mostly about the future. Like it or not you’ll be worried about what’s the next? What will happen? What fate awaits me? What if the pain in my lungs gets worse again in the future; what if this infection causes another problem for my lungs. I got lucky once and survived the disease. There will definitely be no second chance” (Male patient, P2).*

*“At night I was preoccupied with the thought that I’d get worse while sleeping and won’t wake up anymore. I had nightmares and woke up agitated.” (Male patient, P1).*

### A sense of imminent death

In the face of stressors caused by diseases such as increased mortality, patients experienced the feeling of approaching death and being in a life-or-death situation. Participants described their experiences in this regard as follows.

*“The name corona is associated with death. All media you watched talked about death. As the deaths increased, so did my stress. It even killed the youth. When I checked the Internet, I saw pictures of some people younger than me who died. When I saw that this disease did not joke with anyone, I was thinking all the time what if I die!?” (Female patient, P13).*

*“On the second or third day that I lost my sense of smell and taste, I was under very intense stress for a moment, I was scared, I was telling myself what if I sleep at night and do not wake up in the morning; this is called a feeling of imminent death. I felt that I might not be alive an hour later” (Male patient, P4).*

*“During this time, I had a feeling of dangling. I really thought I was suspended between life and death. On days when I was fine, I’d say ‘no, I’d be fine’. The next day I was getting a little worse and I’d say ‘I’m on the verge of death’” (Male patient, P10).*

### Theme 2: psychological distress behind the wall

Based on the participants’ experiences, living in isolation was like living behind the walls, which laid the grounds for psychological distress with a corrosive effect on the mind in them (missing others behind the COVID-19 wall and psychological distress within the family).

### Missing others behind the COVID-19 wall

The dull isolation days and missing the family made patients count the days to get back to normal days. Participants described their experiences in this regard as follows.

*“Isolation days were really boring. It was about a month that I hadn’t visited my parents. I was counting the days until the 14 isolation days were up to return to normal life” (Female patient, P13).*

*“Isolation is difficult for people like me who are active and have a social personality. I felt homesick and bored, especially in the evening, I felt really homesick” (Male patient, P9).*

### Psychological distress within the family

Based on the experiences of participants, the disease and the subsequent isolation created psychological problems among family members (cold atmosphere of the family, the psychological vulnerability of family members, and argument).

One participant said:

*“Our home was not as happy as before. We rarely talked and laughed. Worse was that even what we used to talk and laugh about would bother the family members and even end in quarrels. However, I was never addressed in their quarrels, I knew it was all because of my disease” (Female patient, P8).*

### Theme 3: psychological burden of being a carrier

Stress caused by fear of transmitting the disease, feeling rejected, and COVID-19 stigma were among other psychological disturbances.

### Fear of transmitting the disease

Based on the experiences of the participants, fear of transmitting the disease to family members and worrying about infecting other people put the patient under constant psychological pressure lest they harm any family members, especially children, parents (because of being in high-risk groups) or threaten others. Participants described their experiences in this regard as follows.

*“The greatest fear you have is harming your family. I was afraid of having transmitted the disease to my family, my parents, or my wife” (Male patient, P9).*

*“The stress of infecting people around you is more than that of the disease itself. After infection and having symptoms, my only concern was my family. When I was told I had to be hospitalized, I was in a very bad mood; my mind was preoccupied; I was thinking of my family, especially my daughter who I was worried about being infected.” (Female patient, P7).*

*“While I’m told that I’m 90% better, I’m still really worried, I’m afraid of being a carrier and infecting my daughter or my husband” (Female patient, P12).*

### Feeling rejected

Communication behaviors of others and the medical staff, due to the fear of the possibility of being infected by the patient caused a feeling of being rejected in the patients. Participants described their experiences in this regard as follows.

*“When I greeted two of my colleagues, one of them fled and left, while I have completely recovered!” (Male patient, P1).*

*“The healthcare staff kept away from the patients because they were afraid of getting infected. Some nurses, although being fully equipped, were afraid of coming close and administering an IV fluid or give [me] the pills from a distance. It bothered me” (Male patient, P14).*

### COVID-19 stigma

This concept referred to the negative view and annoying sympathies of others.

Participants described their experiences in this regard as follows.

*“I didn’t step out during these 14 days. Our town is small; everybody knows each other. I was afraid they say he has come out to infect us! Now that I have gone out after 14-15 days, I still have the same feeling. When people see me, they say he has Corona, they run away” (Male patient, P4).*

*“The disease is ill-famed. Everyone who called began crying without saying a word as if I have died or was dying the same day! Out of sympathy, they tried to say things that were as if they are expressing love to us. I sometimes laughed at the way they talk. I was disappointed with their reaction” (Female patient, P11).*

## Discussion

According to studies, COVID-19 patients have a low psychological tolerance capacity, and due to the current status of the disease in the world, these people are highly exposed to psychological disorders [16, 17]. The themes obtained from the data analysis were an attempt to achieve the main goal of the study, i.e. to explore, discover, and describe the psychological disturbances of COVID-19 survivors throughout the disease crisis. Analyzing the interviews and data revealed that psychological disturbances included living in limbo, psychological distress behind the wall, and the psychological burden of being a carrier.

### Living in limbo

Referred to a psychological uncertainty and whether or not they will survive. Confusion or uncertainty due to confiding or not confiding in a wide range of news and information about the disease, is caused by the question of what fate awaits them in the continuation of the disease process, and whether the conditions will worsen and they will get closer to death have caused a sense of struggling between life and death. Jakovljevic et al. reported that COVID-19-related infodemic can be associated with psychological disorders and panic reactions in individuals [18]. Patients may also be confused, anxious, and prone to extreme behaviors [19]. However, due to the COVID-19 pandemic and other epidemics such as SARS, serious concerns are created in such cases, including fear of death among patients due to the unpredictability of the situation and the uncertainty about the time for disease control and seriousness of the danger [20, 21].

### Psychological distress behind the wall

Was described as another psychological disturbance of COVID-19 survivors throughout the disease crisis. During the isolation period, depending on the environmental conditions as well as the type of prevalent infectious diseases, people’s mental health may be impaired and some psychological disorders may occur in isolated people [22]. Patients, who did not know the exact time of the end of the crisis and forced isolation, suffered from psychological disturbances and emotional numbness. In such situations, anxiety can progress to depression, and this is a common risk because the person feels a severe lack of social contact. Isolation causes patients to lose the psychological support of family and friends, which in turn exacerbates stress and psychological trauma. Furthermore, when work and leisure activities are cancelled, the feeling of being locked in, and the intense boredom during isolation at home can irritate the individual and family members, and increase the risk of violence.

### The psychological burden of being a carrier

Imposed a high stress on the patient due to fear of infecting family members and others, feeling rejected, and COVID-19 stigma. Throughout the disease crisis, patients experienced a lot of fear and anxiety regarding the possibility of transmitting the disease and harming family members and others, to the extent that this worry completely troubled their mind.

### The feeling of being rejected

Referred to ways of communication of the healthcare staff and others with the patients. One of the most common reactions of people when they face a COVID-19 patient is fear. Fear is a major feeling that is important for self-defense and survival, and it helps people avoid COVID-19 patients [23, 24]. The same is true for healthcare staff, who may maintain limited contact with patients during the treatment and care period to protect themselves.

Furthermore, the present study found that the negative view and annoying sympathies of others had no result other than the emergence of COVID-19 stigma for patients. Individuals experience episodes of misplaced anger and hostility due to COVID-19 stigma, and feel isolated and rejected. It may be more worrying that stigma harms people’s health and well-being in many ways [25, 26]. Furthermore, stigma groups may be deprived of the resources they need to take care of themselves and their families during an epidemic. On the other hand, the psychological stress caused by stigma can lead to the concealment of the disease in infected people. Thus, the fear of stigma and, consequently, the fear of being labeled, discriminated, rejected, and other such issues lead to concealment and avoiding screening, tests, isolation, and treatment. How to communicate concerning COVID-19 is very important to prevent stigma. An environment must be created in which the disease and its effects are discussed openly, honestly, and effectively [27]. When talking about COVID-19, it should be noted that the language used for addressing these patients should not have a negative connotation or reinforce stigma and labeling attitudes. Using words such as suspicious, isolated, etc. can lead to the continuation of existing negative stereotypes or misconceptions and create widespread fear and panic, or deprive patients of human rights and conditions.

### Limitations

This study only explored the experiences of recovered COVID-19 patients in one of the cities of Iran. However, regarding the nature of the study, generalizing study results is not the qualitative researcher’s concern. Therefore, to increase the generalizability of the results, Studies in this area are recommended to be conducted in other communities. Besides, this was a short-term study.

Long-term involvement with the topic can be a valuable way to identify the psychological disturbances of COVID-19 survivors. The few existing studies regarding mental health problems of COVID-19 survivors limited the discussion and interpretation of the findings, which can also indicate that this study is a pioneer to determine psychological disturbances of COVID-19 survivors throughout the disease crisis.

### Implications of findings

Understanding psychological disturbances of COVID-19 patients can make health officials take measures to alleviate these disturbances via psychological and educational interventions; because although months have passed since the outbreak of the disease, its exact treatment has not been discovered yet, and the number of patients is increasing every day. Therefore, it is better to think of special measures for these patients to relieve them of this psychological burden.

### Future research directions

In the current pandemic, further researches are critically needed to gather more evidence and identify recovered patients who are prone to psychological disorders which can hurt their mental health. So, conducting large-scale longitudinal studies can be helpful in recognizing the psychological effects of recovered COVID-19 patients.

It is also necessary to conduct researches on the implementation of new and effective psycho-educational interventions to reduce the psychological disturbances such as “living in limbo”, “psychological distress behind the wall”, and “psychological burden of being a carrier”. Moreover, educational interventions are required to be implemented at the community level in order to increase the level of awareness and empathy for the population groups at risk of stigmatization.

## Conclusion

The results showed that during pandemics such as the COVID-19 pandemic, in addition to physical and exhausting symptoms of the disease, patients experience disturbances such as living in limbo, psychological distress behind the wall, and the psychological burden of being a carrier. Understanding these disturbances can be helpful in implementing appropriate psychological interventions.

## Data Availability

The datasets used and/or analyzed during the current study are available from the corresponding author on reasonable request.

## Abbreviations

COVID-19: Coronavirus disease 2019
WHO: World Health Organization
SARS: Severe acute respiratory syndrome
MERS: Middle East respiratory syndrome
PTSD: Post-traumatic stress disorder
